# The International Medical Congress of 1869

**Published:** 1869-08

**Authors:** 


THE INTERNATIONAL MEDICAL CONGRESS OF 1869.—The International Medical Congress, which held its first meeting last year, at Paris, will meet this year at Florence, on the 20th September, under the honorary presidency of M. Bouillaud. The work of Congress will be divided as hitherto into two parts—namely, the dicussion of the special questions in the programme, and communications on other medical subjects. The Committee have selected the seven following questions for this year:

1. Marsh miasm; the conditions of its developement in different countries; its effects on man; the curative and preventative remedies.
2. The therapeutic value of the different methods of treating cancerous diseases; their indications and contra-indications; the value of general treatment.
3. The treatment of gunshot wounds, in relation to the progress of the art of war and of modern international law.
4. The hygienic conditions of hospitals, and the value of home treatment.
5. The influence of railways on the health of man.
6. The conditions which favor the production of endemic and epidemic diseases in great towns; the means of prevention, and the advantages to be derived from the proximity of great rivers and seas.
7. The rights and duties of the medical man in relation to the Government in different countries, and the reforms which can be reasonably expected.—*London Lancet.*

